# 조기진통 임부의 불안과 스마트폰 의존도가 수면의 질에 미치는 영향

**DOI:** 10.4069/kjwhn.2020.06.18.2

**Published:** 2020-06-25

**Authors:** Hee Jeong Lee, Hye Young Kim

**Affiliations:** 1 Out-patients Service Team, Keimyung University Dongsan Hospital, Daegu, Korea; 1 계명대학교 동산병원, 외래원무팀; 2 College of Nursing, Keimyung University, Daegu, Korea; 2 계명대학교 간호대학

**Keywords:** Anxiety, Preterm labor, Sleep, Smartphone, 불안, 조기진통, 수면의 질, 스마트폰

## Abstract

**Purpose:**

The purpose of this study was to investigate the effects of anxiety and smartphone dependency on sleep quality in pregnant women with preterm labor.

**Methods:**

The participants of this study were 111 pregnant women who were between 20 and 37 weeks of gestation and experienced preterm labor. The data were collected from October 1, 2018 to October 25, 2019. The collected data were analyzed using descriptive statistics (frequency, percentage, and standard deviation), as well as the t-test, Pearson correlation coefficients, and hierarchical multiple regression.

**Results:**

Significant negative correlations were found between anxiety and sleep quality and between smartphone dependency and sleep quality. Participants’ history of preterm birth, pregnancy method, bowel movements, anxiety, and smartphone dependency significantly affected sleep quality, with an explanatory power of 18%.

**Conclusion:**

In order to improve the quality of sleep, which is an important health-related factor for pregnant women experiencing preterm labor, it will be necessary to identify a history of premature birth, pregnancies achieved using artificial reproductive technology, bowel problems, and smartphone dependency in advance and to provide nursing interventions accordingly.

## Introduction

### 연구 필요성

최근 우리나라 인구 동향 발표에 따르면 전체 출산율은 매년 1명 이하를 기록하며 급격히 감소하고 있지만, 고위험 신생아인 조산아 출산율은 지속해서 증가하는 양상을 보이고 있으며, 전체 출산율에서 조산율은 1997년 3.3%에서 2015년 6.7%로 지속적으로 증가하고 있다[1].

조산의 주요 원인을 차지하는 조기진통은 임신 20주에서 37주 사이에 자궁경부의 진행적 변화인 개대와 소실이 동반되면서 규칙적으로 자궁 수축이 있는 경우를 말한다[2]. 조기진통으로 진단받고 진료를 받은 임부 수는 2015년 38,671명, 2016년 43,052명, 2017년 47,469명으로 꾸준한 증가 추세에 있는데, 고령 초산 임부의 비율이 증가하고 여성의 사회활동이 늘면서 이로 인한 정신적, 육체적 스트레스가 조산의 원인으로 보고되고 있다[3,4].

조기진통을 진단받은 임부는 임신 유지를 위해 병원에 입원하여 증상을 관찰하고 침상 안정, 진통 억제제와 같은 약물 치료를 받는다[5]. 특히 조기진통 임부의 침상 안정은 가장 빈번하게 시행하는 치료적 관리 및 간호 중 하나로, 이는 신체활동을 제한하여 조기 분만을 예방하고 태아가 자궁 내에서 성장할 수 있도록 유지하여 고위험 분만의 위험을 감소시키는 것이다[6]. 조기진통 임부의 침상 안정은 임신 유지를 위해 필수적이지만, 활동을 엄격히 제한하는 침상 안정은 임부의 심리적, 신체적 건강에 부정적인 영향을 미칠 수 있다[7].

조기진통 임부는 임신 유지, 출산 및 태아의 상태에 대한 불확실성으로 인해 불안을 느끼고 있으며, 입원으로 인한 경제적 부담감, 가족과의 분리 등으로 인해 부정적인 상태를 경험한다[8]. 장기간의 침상 안정으로 인해 심리적 불안감을 경험하는 조기진통 임부는 불안 해소와 지식 획득을 위해 자신의 상태를 의료진과 상담하기보다 접근이 용이한 인터넷을 통해 자신의 상황과 유사한 경험담을 보고 자신의 상태를 예측하며 임신에 관한 지식을 습득한다[9,10]. 조기진통 임부들이 정보 탐색 경로로 사용하는 스마트폰은 여러 컴퓨터 지원 기능을 추가한 지능형 단말기로, 시간과 장소에 제한되지 않고 인터넷을 이용할 수 있는 장점이 있다. 그러나 휴대성이 좋고 언제 어디서든 쉽게 사용할 수 있는 특성으로 인해 스마트폰 과의존이라는 문제점도 증가하고 있다[11]. 스마트폰 사용 실태 조사에 따르면 여성이 남성보다 고위험군과 잠재적 위험군 비율이 높게 나타났는데, 20대는 고위험군과 잠재적 위험군이 22.3%, 30대는 고위험군과 잠재적 위험군이 15.8%로 각각 조사되었다[12].

스마트폰 의존도가 높을수록 정신적, 신체적 부작용이 나타나며 수면의 질에 영향을 미친다. 스마트폰 과의존으로 인해 잠드는 데 오랜 시간이 걸리고, 밤에 자주 깨거나 이른 시간 기상, 주간 졸림과 같은 수면 장애가 나타나는 것으로 보고되고 있다[13]. 또한 스마트폰 화면에서 나오는 밝은 빛은 수면을 유도하는 호르몬인 멜라토닌 분비를 저하시켜 수면 장애를 가져온다[14]. 임부에게 있어 수면은 신체 기능을 회복하고 건강을 유지하기 위한 필수적 요소로, 임부의 적절한 수면은 정신적 상태에 영향을 미친다[15]. 그러나 임신 동안 나타나는 호르몬 변화와 생리적 변화는 수면 장애로 이어질 수 있는데, 임부의 경우 수면 장애 발생률이 60.0%로 비임부의 48.0%보다 높다. 임신 기간 중 충분한 수면을 취하지 못할 경우 임부와 태아의 건강 상태에 영향을 미치는데, 조산을 높이고 분만 시간을 연장시킨다[16]. 따라서 임부의 수면 질은 건강한 임신 결과를 위해 중재되어야 할 중요한 건강문제이다.

조기진통 임부가 정보 획득을 위해 스마트폰을 가장 많이 활용하고 있다는 연구가 있음에도 불구하고[9] 조기진통 임부를 대상으로 한 선행 연구는 조기진통 임부의 상태 불안과 정서적 상태[10], 조기진통 임부의 신체적 요구도[8], 조기진통 임부의 침상 경험[9], 조기진통 임부의 간호 요구도[17]에 대한 연구로 한정되어 간호학에서 조기진통 임부의 스마트폰 의존도와 수면의 질에 관계된 연구는 없었다. 스마트폰 사용인구가 20–30대 여성층에서 높은 비중을 차지하고 있고, 부모가 스마트폰 고위험군일 경우 영유아 자녀도 고위험군에 포함될 비율이 높기 때문에[12], 조기진통 임부의 스마트폰 의존도에 대한 평가와 모니터링이 건강에 미치는 영향 등에 대해 알아보는 것은 매우 중요하다 하겠다.

따라서 본 연구는 조산의 주요 원인 중 하나로 알려진 조기진통 임부들의 일반적, 산과적 특성과 함께 불안, 스마트폰 의존도와 수면의 질을 파악하고 이를 통해 조기진통 임부의 스마트폰 의존 발생 예방 및 관리, 수면의 질 제고에 도움이 될 수 있는 기초 자료를 제공하고자 시도되었다.

### 연구 목적

본 연구는 조기진통 임부의 불안, 스마트폰 의존도가 수면의 질에 미치는 영향을 파악하는 데 목적이 있으며, 구체적인 목적은 다음과 같다.

1) 대상자의 일반적, 산과적 특성에 따른 수면의 질 정도를 파악한다.

2) 대상자의 불안, 스마트폰 의존도와 수면의 질과의 상관관계를 파악한다.

3) 대상자의 수면의 질에 영향을 미치는 요인을 파악한다.

## Methods

Ethics statement: This study was approved by the Institutional Review Board (IRB) of Keimyung University (IRB. No. 40525-201807-HR-66-05). Informed consent was obtained from the participants.

### 연구 설계

본 연구는 조기진통 임부의 불안, 스마트폰 의존도와 수면의 질 정도를 확인하고, 조기진통 임부의 수면의 질에 영향을 미치는 요인을 파악하고자 하는 상관성 조사연구 설계를 이용하였다.

### 연구 대상

본 연구의 대상자는 대구광역시 소재 계명대학교 동산병원 산부인과 병동에 조기진통으로 진단받고 입원 중인 임부를 대상으로 하였다. 연구 대상 선정 기준은 조기진통을 주 진단으로 입원한 임신 20주에서 37주 미만의 임부이고, 본 연구의 목적을 이해하고 참여를 하고 설문지 내용을 이해하고 응답할 수 있는 임부를 포함하였다. 정신과적 질환을 진단받은 경험이 있거나, 현재 정신과적 약물을 복용하는 임부복용하거나 신체적 장애 및 기타 장애로 설문지를 읽고 답할 수 없는 임부와 결혼이주여성은 제외하였다.

표본의 크기를 결정하기 위해 G*Power 3.1.9.2 프로그램을 사용하여 선행 연구[15]에서 제시한 유의수준 .05, 중간 정도의 효과크기(f2) .20, 검정력 .80을 기준으로 예측변수 17개를 투입했을 때 최소한의 표본수 104명이 산출되었다. 탈락률을 고려하여 120명에게 자료 수집하였으며, 수집된 자료 중 불성실한 답변을 한 9명의 자료는 제외하여 최종 111명의 자료를 분석하였다.

### 연구 도구

#### 불안

불안을 측정하기 위해 Spielberger [18]의 State Trait Anxiety Invention-Y형(STAI-Y)을 Han 등[19]이 한국어로 번안한 상태 불안 관련 20문항을 번안자의 승인을 받아 사용하였다. 상태 불안 관련 20문항은 염려, 긴장, 초조, 걱정 등에 대해 지금 이 순간에 바로 느끼고 있는 상태를 측정하며, 긍정 문항과 부정 문항이 각 10개씩 구성되어 있다. ‘전혀 그렇지 않다’, ‘그렇지 않다’, ‘그렇다’, ‘매우 그렇다’까지 Likert 4점 척도로 응답하게 되어 있다. 긍정 문항은 역 환산 처리했다. 총점 범위는 20점에서 80점까지로 점수가 높을수록 상태 불안 정도가 높음을 의미한다. 도구의 신뢰도는 Han 등[19]의 연구에서는 Cronbach’s α값 .92였으며 본 연구에서 Cronbach’s α값은 .93이었다.

#### 스마트폰 의존도

Kim 등[20]이 개발한 성인 스마트폰 의존도 자가진단척도(S-척도)를 원저자의 승인을 받아 사용했다. 스마트폰 의존도 척도는 총 15문항으로, 하위척도는 일상생활 장애 5문항, 가상세계 지향성 2문항, 금단 4문항, 내성 4문항의 네 가지 하위요인으로 구성되어있다. ‘전혀 그렇지 않다’, ‘그렇지 않다’, ‘그렇다’, ‘매우 그렇다’까지 Likert 4점 척도이며 총점 범위는 15점에서 60점까지로 점수가 높을수록 스마트폰 의존 성향이 높음을 의미한다. 도구의 신뢰도는 Kim 등[20]의 연구에서는 Cronbach’s α값이 .81이었으며, 본 연구의 Cronbach’s α값은 .86이었다.

#### 수면의 질

수면의 질은 Oh 등[21]이 개발한 측정 도구를 원저자의 승인을 받아 사용했으며 총 15문항으로 하위척도는 수면 양상 7문항, 수면 평가 4문항, 수면 결과 2문항, 수면 저해요인 2문항으로 구성되어 있다. ‘전혀 그렇지 않다’, ‘그렇지 않다’, ‘그렇다’, ‘매우 그렇다’까지 Likert 4점 척도로 응답하게 되어 있다. 긍정문항 2개는 역 환산 처리하였다. 총점 범위는 15점에서 60점까지로 점수가 높을수록 수면의 질이 높은 것을 의미한다. 도구의 신뢰도는 Oh 등[21]의 연구에서는 Cronbach’s α값이 .75였으며 본 연구의 Cronbach’s α값은 .89로 나타났다.

### 자료 수집

자료 수집은 2018년 10월 1일부터 2019년 10월 25일까지 대구광역시 소재 계명대학교 동산병원에서 조기진통 임부를 모집하였으며 입원 병동에 모집공고문을 게시하여 자율적 참여의사를 밝힌 임부에게 연구자가 직접 연구의 목적을 설명하고 설문지 배부 하였다. 회수는 밀봉상태로 자유롭게 제출하여 연구자가 수거하였고 답례로 소정의 상품을 제공하였다. 총 120명의 대상자에게 설문조사를 진행하여 불성실한 답변을 제외한 111명의 자료를 분석하였다.

### 자료 분석

본 연구에서 수집된 자료는 IBM SPSS Statistics for Windows ver. 24.0 (IBM Corp., Armonk, NY, USA)을 이용하여 분석했다.

대상자의 특성을 알아보기 위해 평균, 백분율, 표준편차 등의 기술통계를 실시했다. 대상자의 불안, 스마트폰 의존도와 수면의 질을 파악하기 위해 평균 및 표준편차로 분석하였고, 대상자의 특성에 따른 수면의 질 정도의 차이는 t-test로 분석하였다. 대상자의 불안, 스마트폰 의존도와 수면의 질 간의 상관관계를 파악하기 위해 상관관계 분석(Pearson correlation coefficient)을 하였으며, 대상자의 수면의 질에 영향요인을 확인하기 위해 위계적 회귀분석(hierarchical multiple regression)을 하였다.

## Results

### 대상자의 특성

대상자의 평균 나이는 33.58세였으며, 35세 이상인 경우가 42.3%였고, 대학교를 졸업한 대상자가 57.7%였다. 하루 평균 수면시간은 7.49시간, 하루 평균 스마트폰 사용 시간은 5.71시간으로 스마트폰 사용 주요 동기는 83.1%였다. 평균 임신 기간이 28주 이상이 58.6%였고, 조산 경험이 있는 대상자는 14.4% 하였고 규칙적으로 산전진찰을 받는 대상자는 70.3%를 차지하였다. 유산 경험이 있는 대상자는 43.2%였다. 자연 임신의 비율이 66.7%로 나타났다(Table 1).

### 대상자의 불안과 스마트폰 의존도 및 수면의 질

대상자의 불안과 스마트폰 의존도, 수면의 질 측정과 관련된 결과는 Table 2와 같다. 불안은 총점 80점 만점에 평균 43.43±9.50점이며 최소값은 21, 최대값은 72로 나타났다. 스마트폰 의존도 점수는 총점 60점에 평균 36.50±7.84점이며 최소값은 21점, 최대값은 55점이었다. 수면의 질 점수는 총점 60점에 평균 38.42±8.53점이었고, 최소값 20점, 최대값 58점으로 확인되었다(Table 2).

### 대상자의 특성에 따른 수면의 질 차이

대상자의 일반적 특성에 따른 수면의 질을 검증한 결과에서 대상자의 산과적 특성으로 조산경험여부(t=-2.06, *p*=0.042), 임신유형(t=-2.54, *p*=.012), 배변양상(t=-2.26, *p*=.026)에 따라서 통계적으로 유의한 차이가 있게 나타났다(Table 3).

### 대상자의 불안, 스마트폰 의존도와 수면의 질 상관관계

대상자의 불안, 스마트폰 의존도와 수면의 질과의 상관관계를 확인한 결과, 대상자의 수면의 질과 불안은 통계적으로 유의한 음의 상관관계를 보였다(r=–.23, *p*=.013). 수면의 질과 스마트폰 의존도 역시 유의한 음의 상관관계가 있었다(r=–.24, *p*=.010). 즉, 대상자의 불안이 낮을수록, 스마트폰 의존도가 낮을수록 수면의 질이 높아짐을 알 수 있었다.

### 대상자의 수면의 질에 영향을 미치는 요인

대상자의 일반적, 산과적 특성과 스마트폰 의존도, 불안이 수면의 질에 미치는 영향을 분석한 결과는 Table 4와 같다.

회귀분석 실시 조건을 확보하기 위해 종속변수의 자기 상관과 독립변수 간 다중공선성을Durbin-Watson 지수와 분산팽창지수(variance inflation factor, VIF)를 이용하여 검토했다. 그 결과 Durbin-Watson 지수는 1.557로 2에 가까워 자기 상관이 없었으며, VIF는 1.012–1.068의 10 미만으로 독립변수 간의 다중공선성은 존재하지 않는 것으로 나타났다.

조기진통 임부의 불안, 스마트폰 의존도가 수면의 질에 미치는 영향요인을 파악하기 위해 일반적 특성에 유의한 차이를 보인 수면시간, 배변 양상, 조산 유무, 임신 방법을 독립변수로 투입하여 회귀분석을 실시한 결과는 다음과 같다. 독립변수 중 명목변수는 더미변수로 처리하였다. 먼저 통제변수인 대상자의 일반적 특성을 투입한 모형 1의 경우, 대상자의 일반적 특성 가운데 배변 양상 중 정상일수록(t=2.57, *p*=.011), 임신 방법 중 자연 임신일 경우(t=–2.68, *p*=.008)와 조산 경험이 없는 경우(t=–2.41, *p*=.017) 수면의 질에 유의한 영향을 미치는 것으로 나타났다. 모형 1의 설명력은 14%로 분석되었다(F=5.50, *p*<.001). 모형 1에 투입된 변수들을 통제한 모델 2에서 불안, 스마트폰 의존도를 추가로 투입한 경우 회귀모형이 유의하였으며 모형 2의 설명력은 18%로 분석되었다(F=5.23, *p*<.001). 그러나 개별변수 불안에서는 유의성이 나타나지 않았고(β=–.12, *p*=.165), 스마트폰 의존도가 높을수록(β=–.19, *p*=.028) 수면의 질에 유의한 영향을 미치는 것으로 나타났다.

## Discussion

본 연구는 조산의 주요 원인 중 하나로 알려진 조기진통을 경험하고 있는 임부들의 일반적, 산과적 특성과 함께 조기진통 임부의 불안과 스마트폰 의존도가 수면의 질에 미치는 영향을 파악하고 이를 통해 조기진통 임부의 불안과 스마트폰 과의존 예방 및 수면의 질 제고에 도움이 되고자 수행하였으며, 그 결과에 대한 논의는 다음과 같다.

본 연구에서 조기진통 임부의 상태 불안 점수는 총점 80점에 평균 43.43점이었다. 이는 조기진통 임부를 대상으로 동일한 도구를 사용한 연구[22]에서 나타난 상태 불안 점수 42.8점보다 높았다. 정상 임부를 대상으로 본 연구와 동일한 도구를 사용한 연구[23]에서는 불안 정도가 39.30점으로 나왔다. 이러한 결과를 통해 본 연구의 대상자의 상태 불안 점수가 더 높음을 알 수 있다. 조기진통 임부는 조산에 대한 불안감과 이로 인한 태아 상태에 대한 걱정으로 정상 임부보다 불안이 더 높은 것으로 생각되며, 조기진통 임부에 대한 불안 사정의 중요성과 불안을 완화할 수 있는 간호 중재가 필요할 것으로 판단된다.

본 연구에서 조기진통 임부의 스마트폰 의존도 점수는 총점 60점에 평균점수는 36.5점이었다. 조기진통 임부나 입원 환자를 대상으로 한 스마트폰 과의존 선행 연구는 없었으나 대학생들을 대상으로 동일한 측정 도구를 사용한 Jeon과 Jang [24]의 연구에서 나타난 스마트폰 의존도 평균 점수 35.7점보다 높게 측정되었다. 이러한 결과는 스마트폰 과의존 수준이 가장 높은 집단으로 볼 수 있는 대학생보다도 높게 나온 것으로, 조기진통 임부의 경우 스마트폰을 이용해 자신의 질병에 대한 정보를 얻거나 또는 같은 질병을 가진 커뮤니티와 소통을 위한 수단으로 활용하고 있기 때문으로 생각된다. 특히 조기진통 임부가 입원 중 일상적인 활동을 제한받고 절대 안정을 취하는 상황에서 접근이 용이한 스마트폰에 많이 의존함을 알 수 있다. 이러한 결과를 통해 조기진통 임부를 대상으로 스마트폰을 활용하여 건강 관련 정보 및 태교, 복식호흡 훈련 등을 제공할 수 있는 간호 중재가 마련될 필요가 있을 것이다.

본 연구에서 조기진통 임부의 수면 점수는 총점 60점 만점에 38.42점으로 측정되었다. 이러한 점수는 동일한 측정 도구를 사용한 유방암 환자 대상 연구[25]의 수면 점수 38.85점과 유사한 결과이다. 즉 조기진통 임부의 수면의 질이 유방암 환자의 수면의 질과 비슷함을 알 수 있다. 이러한 결과에 대해 조기진통 임부는 입원으로 인한 소음, 야간 처치와 같은 환경적 요인과 신체적 요인, 불안과 같은 감정적 요인으로 인해 수면의 질이 떨어지는 것으로 추정할 수 있다. 조기진통 임부의 수면의 질을 높이기 위해 안정적인 병실 환경을 조성하고, 심리적 불안감과 신체적 불편감을 낮추기 위한 간호 중재를 마련할 필요가 있다.

조기진통 임부의 불안과 스마트폰 의존도, 수면의 질과의 상관관계를 분석한 결과 조기진통 임부의 스마트폰 의존도와 수면은 음의 상관관계를 보였는데, 이는 스마트폰 의존도가 높은 임부는 수면의 질이 낮음을 의미한다. 이는 선행 연구에서 스마트폰 의존도가 높을수록 신체 활동량이 줄고 수면의 질이 낮아진다는 보고 결과와도 일치한다[26]. 조기진통 임부들은 대부분 조산 방지를 위해 입원 치료를 시행하고 침상 안정으로 활동이 제한되어 있기 때문에 스마트폰을 과다하게 사용하는 경향이 있는데, 스마트폰 과의존으로 인해 수면의 질이 낮아질 수 있다. 따라서 높은 스마트폰 의존 경향을 가진 조기진통 임부들에게 적절한 스마트폰 사용 방법과 시간에 대한 교육이 이루어진다면 수면의 질 수준을 높이는 데 기여할 수 있을 것이다.

조기진통 임부의 불안은 수면과 음의 상관관계를 보였다. 이 결과는 조기진통 임부들의 현상학적 경험을 보고한 연구[10]에서 조기진통 임부들은 조산에 대한 불안감과 두려움으로 인해 밤에 깊은 잠을 자지 못하고, 병원의 물리적인 환경으로 인해 수면에 불편을 호소한다는 결과와 유사했다. 따라서 조기진통 임부의 불안을 낮추기 위해 불안의 원인을 찾고 의학 지식에 의한 정확한 정보를 의료진이 전달하며, 편안한 병원 환경을 만들어 불안을 낮추고 수면을 증진할 수 있는 간호 중재안이 마련될 필요가 있다.

본 연구에서 조기진통 임부의 수면의 질에 영향을 미치는 요인을 파악하기 위해 단계적 다중 회귀분석을 하였고 그 결과 임신 방법, 조산 유무, 배변 양상, 스마트폰 의존도가 수면의 질의 영향요인으로 나타났으며, 수면의 질을 총 18% 설명하는 것으로 나타났다.

수면의 질에 영향을 미치는 요인은 임신 방법과 조산 유무였다. 자연 임신은 수면의 질에 부적 영향을 미치며, 과거의 조산 경험은 수면의 질에 정적인 영향을 미쳤다. 이와 같은 결과는 조기진통 임부의 과거 조산 경험이 수면 장애의 위험을 증가시킨다는 연구 결과와 차이를 보였다[27]. 하지만 최근 난임 여성을 대상으로 수면의 질을 연구한 선행 연구에서 난임 여성들에게 충분한 수면 시간과 수면의 질은 임신율에 영향을 미친다고 보고하여 난임 여성들의 수면이 중요함을 알 수 있다[28]. 본 연구 결과 조산 경험이 있고 보조생식술을 시행한 대상자 군에서 평균 7시간 이상 수면시간을 갖는 것으로 추측되며, 이러한 수면 시간이 수면의 질과 연관이 있다고 생각되나 추후 이에 대한 반복 연구가 필요할 것이다.

두 번째로 수면의 질에 영향을 미치는 요인은 배변 양상이었다. 규칙적인 배변 양상은 수면의 질에 정적인 영향을 미쳤다. 이와 같은 연구 결과는 임부가 느끼는 신체적 불편감 중의 하나인 변비나 설사와 같은 불규칙적 배변 양상이 수면 장애와 연관이 있다는 선행 연구 결과와 유사하였다. 임부는 임신 중 호르몬의 변화와 장 운동의 감소 및 임신 중 영양 요구 사항을 충족시키기 위한 다량의 단백질과 지방 섭취로 인해 변비 발생률이 높으며 변비로 인해 수면의 질이 떨어진다[27,29]. 이와 같은 연구 결과를 바탕으로 조기진통 임부에게 가벼운 산책이나, 운동을 권유하고 단백질 섭취시 식이섬유도 함께 섭취할 것을 교육하면 변비를 예방하고 수면의 질을 높일 수 있을 것이다.

마지막으로 수면의 질에 영향을 미치는 요인은 스마트폰 의존도였다. 스마트폰 의존은 수면의 질에 부적 영향을 미쳤다. 이와 같은 결과는 Heo 등[30]의 연구에서 스마트폰 과의존이 수면의 질에 유의한 영향을 미치는 것으로 확인된 것과 일치한다. 특히 취침 전에 스마트폰을 사용하는 것은 수면의 질을 악화시키는 요인으로, 스마트폰 화면에서 나오는 밝은 빛은 수면을 유도하는 호르몬인 멜라토닌 분비를 저하해 수면 장애를 가져온다[14]. 이와 같은 연구 결과를 바탕으로 조기진통 임부의 스마트폰 이용 동기를 파악하고, 적절한 스마트폰 사용 지침과 취침 전 스마트폰 사용은 수면에 방해가 된다는 것을 교육하여 수면의 질을 높일 수 있을 것이다.

본 연구에서 불안은 수면의 질과 유의한 상관관계가 있으나, 수면의 질에 단독으로 영향을 미치는 요인으로 확인되지는 않았다. 이는 조기진통 임부의 정서적 상태인 불안이 수면과 상관관계가 있음을 나타내는 연구와 차이를 보인 결과이다[8]. 본 연구에서 조기진통 임부들이 불안을 해소하기 위한 대처 기전으로 스마트폰을 사용하였다면 오히려 두 변수 사이에 매개변수로 작용하여 본 연구 결과에 영향을 미칠 수 있을 것으로 생각되며, 불안이 수면의 질에 미치는 영향에 대해서는 추후 반복 연구가 필요할 것이다.

이상과 같이 본 연구에서는 조기진통 임부의 수면의 질에 영향을 미치는 요인들, 즉 조산 유무, 임신 방법, 배변 양상과 스마트폰 의존도가 수면의 질에 유의한 영향이 있는 것으로 나타났다. 이러한 여러 요인을 종합하여 임신 시 수면에 어려움을 겪을 고위험 요인들에 노출된 임부들을 선정하고 이들에 대한 교육 및 관리가 체계적으로 이루어진다면 조기진통 임부의 수면의 질을 높이는 데 도움이 될 것이라 생각된다. 다만 본 연구는 연구자가 편의 추출한 단일 지역의 단일 대학병원에서 진행하였기 때문에 연구 대상자가 한정되어 연구 결과를 일반화하기에는 어려움이 있으며, 변수와의 관계에서 효과를 검증하지 못한 제한점이 있다. 스마트폰 의존도가 조기진통 임부의 수면의 질 이외에도 조기진통 임부의 스트레스와 출산 결과에 어떠한 영향을 미치는지에 대한 추후 연구를 제언한다.

## Figures and Tables

**Table 1.** Characteristics of the participants (N=111)

Characteristic	Categories	n (%) or Mean±SD
General characteristic		
Age (year)		33.58±4.38
	< 35	64 (57.7)
	≥35	47 (42.3)
Job	Yes	49 (44.1)
	No	62 (55.9)
Religion	Yes	50 (45.0)
	No	61 (55.0)
Years of education (year)	<16	47 (42.3)
	≥16	64 (57.7)
Family income (×10,000 KRW/month)	<400	55 (49.5)
	≥400	56 (50.5)
Sleeping hours (/day)		7.49±1.72
	<7	35 (31.5)
	≥7	76 (68.5)
Smartphone use (hour/day)		5.71±3.46
Major reason for using a smartphone^†^	Searching information	103 (83.1)
	SNS	5 (4.0)
	Games	9 (7.3)
	Business activities	7 (5.6)
Obstetric characteristics		
Gestational age (week)	20–27	46 (41.4)
	28–37	65 (58.6)
History of preterm labor	Yes	16 (14.4)
	No	95 (85.6)
Family history of preterm labor	Yes	14 (12.6)
	No	97 (87.4)
Prenatal examinations	Regular	78 (70.3)
	Irregular	33(29.7)
History of abortion	Yes	48 (43.2)
	No	63 (56.8)
Type of pregnancy	ART	37 (33.3)
	Spontaneous	74 (66.7)
Bowel movements	Regular	67 (60.4)
	Irregular	44 (39.6)

ART: assisted reproductive techniques; KRW: Korean won; SNS: social networking services.

† Multiple responses.

**Table 2.** Anxiety, smartphone dependency, and sleep quality of the participants (N=111)

Variable	Range	Mean±SD
Anxiety	21–72	43.43±9.50
Smartphone dependency	21–55	36.50±7.84
Sleep quality	20–58	38.42±8.53

**Table 3.** Differences in sleep quality according to participants’ characteristics (N=111)

Characteristic	Categories	Mean±SD	t	*p*
General characteristic				
Age (year)	< 35	38.27±7.94	–0.22	.821
	≥35	38.64±9.37		
Job	Yes	40.14±9.08	–1.9	.059
	No	37.06±7.89		
Religion	Yes	39.14±8.11	0.79	.426
	No	37.84±8.89		
Years of education (year)	<16	37.34±8.33	–1.14	.254
	≥16	39.22±8.66		
Family income (×10,000 KRW/month)	<400	37.45±8.25	1.18	.238
	≥400	39.38±8.77		
Sleeping hours (/day)	<7	35.37±8.00	–2.62	.01
	≥7	39.83±8.45		
Obstetric characteristic				
Gestational age (week)	20–27	39.22±8.16	0.82	.412
	28–37	37.86±8.81		
History of preterm labor	Yes	42.44±8.47	–2.06	.042
	No	37.75±8.40		
Family history of preterm labor	Yes	37.50±9.18	0.43	.667
	No	38.56±8.48		
Prenatal examinations	Regular	39.15±8.75	1.39	.167
	Irregular	36.70±7.85		
History of abortion	Yes	39.63±8.71	–1.29	.197
	No	37.51±8.35		
Type of pregnancy	ART	41.27±8.90	2.54	.012
	Spontaneous	37.00±8.03		
Bowel movements	Regular	39.88±8.95	–2.26	.026
	Irregular	36.20±7.42		

ART: assisted reproductive technology; KRW: Korean won; SNS: social networking services.

**Table 4.** Factors affecting the sleep quality of the participants (N=111)

Variable	Model 1				Model 2			
	B	β	t	*p*	B	β	t	*p*
(Constant)	33.1		8.94	<.001	45.99		7.78	<.001
Sleep hours	0.67	0.13	1.51	.133	0.66	0.13	1.53	.127
Bowel movements (regular=1)	3.99	0.23	2.57	.011	3.31	0.19	2.15	.034
History of preterm labor (no=1)	–5.19	–.21	–2.41	.017	–4.75	–.19	–2.26	.026
Method of pregnancy (spontaneous=1)	–4.31	–.23	–2.68	.008	–3.92	–.21	–2.5	.014
Anxiety					–0.11	–.12	–1.39	.165
Smartphone dependency					–0.21	–.19	–2.22	.028
	R^2=^.17, adjusted R^2^=.14, F=5.50,* p*<.001				R^2=^.23, adjusted R^2^=.18, F=5.23, *p*<.001			
